# The German day-care study: multicomponent non-drug therapy for people with cognitive impairment in day-care centres supplemented with caregiver counselling (DeTaMAKS) – study protocol of a cluster-randomised controlled trial

**DOI:** 10.1186/s12913-017-2422-x

**Published:** 2017-07-17

**Authors:** Elisa-Marie Behrndt, Melanie Straubmeier, Hildegard Seidl, Stephanie Book, Elmar Graessel, Katharina Luttenberger

**Affiliations:** 1 Department of Psychiatry and Psychotherapy, Centre for Health Services Research in Medicine, Friedrich-Alexander-University Erlangen-Nürnberg (FAU), Schwabachanlage 6, 91054 Erlangen, Germany; 2 Institute of Health Economics and Health Care Management, Helmholtz Zentrum, München, Ingolstädter Landstraße 1, 85764 Neuherberg, Germany

**Keywords:** Day-care, Multimodal intervention, Non-drug, Dementia, MCI, Informal caregivers

## Abstract

**Background:**

It is the wish of both people with cognitive impairment and their informal caregivers for the impaired person to live at home for as long as possible. This is also in line with economic arguments about health. The existing structure of day-care services for the elderly can be used to achieve this. Due to the current lack of empirical evidence in this field, most day-care centres do not offer a scientifically evaluated, structured intervention, but instead offer a mixture of individual activities whose efficacy has not yet been established. Informal caregivers of people with dementia use day-care centres primarily to relieve themselves of their care tasks and as a support service.

**Methods/design:**

The present study therefore investigates the effectiveness of a combination of a multicomponent activation therapy for people with mild cognitive impairment (MCI) or mild to moderate dementia at day-care centres and a brief telephone intervention for their informal caregivers. The study is conducted as a cluster-randomised intervention trial at 34 day-care centres in Germany with a 6-month treatment phase. The centres in the waitlist control group provide “care as usual”. A power analysis indicated that 346 people should initially be included in the study. The primary endpoints of the study include the ability to perform activities of daily living (ADL) and cognitive capacities on the side of the day-care centre users and the subjectively perceived burden and well-being of the informal caregivers. The total duration of the study is 3 years, during which data are collected both by the psychometric testing of the people with cognitive impairment and by telephone interviews with informal caregivers.

**Discussion:**

The project has three distinctive quality features. First, it is embedded in real care situations since the day-care services have already been established for this target group. Second, due to the large number of cases and the fact that the participating day-care centres are spread across the entire country, the results can be expected to be generalisable. Third, the interventions can be assumed to be implementable as they required only a one-day training event for the staff already working at the centres.

**Trial registration:**

ISRCTN16412551 (Registration date: 30 July 2014, registered retrospectively).

## Background

Both elderly people with cognitive impairment and their informal caregivers often have the same concern – that care recipients should remain in their own homes for as long as possible [1]. In addition to the personal needs of the individuals in question, the healthcare system also has an interest in the economic advantage of keeping elderly people with MCI and dementia at home [2].

In order to achieve this in people with MCI or dementia, it is necessary to help them remain independent for as long as possible and to reduce the burden experienced by their informal caregivers. To date, no effective interventions have been developed, evaluated, or implemented in the existing outpatient care services.

One institution that is already in place in the current healthcare system and in a position to reach dyads consisting of informal caregivers and people with MCI or dementia is the day-care centre. In Germany, these centres have already been established as providers of services for people requiring care, thus relieving the burden on informal caregivers [3]. They also receive funding from the statutory care insurance.

Services provided by day-care centres in Germany are currently used by only a small percentage (approx. 4%) of people needing care who are cared for at home [4]. The majority of the day-care centre users are elderly people with varying levels of cognitive deficits – from MCI to severe dementia [5]. In people with Alzheimer’s dementia [6], there is an association between the attendance of a day-care centre and the transition to institutionalised care.

If a person requiring care attends a day-care centre, this directly relieves the care burden on their informal caregiver. It also reduces depressive mood in the caregivers [7]. Moreover, the people requiring care who attend a day-care centre experience an increase in well-being [8].

However, the results of a recent review revealed that, to date, there is no scientifically tested, established intervention concept [8] with concrete goals and a structured manual for people with cognitive impairment who attend day-care centres. Such a development could transform these centres into a form of specific intervention rather than just care as usual. This means that in the past, day-care centres have not fully exploited their capabilities to promote attendees’ independence and to contribute to stabilising the attendees’ abilities to live at home. It is possible for day-care centres to carry out a non-drug group therapy with only minimal additional effort, using their existing staff and premises. It is also less expensive than an individual intervention at home.

The fact that the nursing staff of day-care centres can reach both people with cognitive impairment and their caregivers, a multicomponent intervention that supports both would be useful. Multicomponent interventions are also more effective than single interventions, for both people with dementia [9] and their caregivers [10].

The scientific evidence on non-drug treatments for dementia is mixed, and it is therefore not possible to make an unreserved recommendation [11]. The evidence on the therapeutic benefit of pharmacotherapeutic treatment options for Alzheimer’s dementia is also limited, particularly with regard to the duration of effect, adverse effects, and clinical relevance [12]. There are indications that occupational group training improves ADL, but it has no significant effect on cognitive abilities [13]. These results clearly indicate the need to develop and test effective interventions and especially multicomponent interventions in order to achieve effects in several domains of function.

In this situation, the non-drug multicomponent treatment “MAKS” (standing for **m**otor stimulation, **a**ctivities of daily living, and **c**ognitive and **s**ocial functioning) [14] was developed and tested in a randomised-controlled intervention study in an inpatient setting for a duration of 12 months [15, 16]. It resulted in a statistically significant and clinically relevant therapeutic effect in which the capacities of the people with dementia were stabilised in the domains of ADL and cognitive function. The treatment effect was also sustained over a follow-up period of 10 months.

Caregiver burden also results from a progressive decline in the capacity to perform ADL in people with dementia [17]. Thus, an intervention that has a favourable influence on the ability to perform ADL also has an indirect effect on the burden experienced by caregivers by slowing down the increase in activities they have to take on for the person requiring care.

“Unexplainable” or challenging behaviours of people with dementia also play a special role in the burden experienced by informal caregivers [18]. Telephone support for caregivers of people with dementia can be helpful because it improves their emotional health [19]. A combination of day-care for people with cognitive impairment and telephone support for their informal caregivers may be more effective than separate interventions [20].

Thus, we combine a non-drug therapy for people with cognitive impairment in the day-care setting with caregivers’ support by offering a low-threshold counselling service. For this purpose, the MAKS treatment, which has already demonstrated effectiveness in the nursing home setting, was adjusted for the day-care setting.

The acceptance of both approaches – the multimodal, non-drug MAKS therapy [15] and the telephone interventions for caregivers [21] – has been demonstrated in previous studies and can be considered high.

The objective of this paper is to describe the study protocol of the cluster-randomised DeTaMAKS (**De**menz – **Ta**gespflege – **MAKS**; engl. **de**mentia – **da**y-care – **MAKS** therapy) study and to serve as a reference for forthcoming papers reporting the results of the study.

## Methods/design

### Aims and hypothesis

The main aim of the DeTaMAKS study, which began in October 2014, is to assess whether a combined intervention can promote and sustain abilities to perform ADL and cognitive capacities and thus independence in people with MCI or mild or moderate dementia, which should also provide some relief for the informal caregiver.

There is also the hope that combining the MAKS therapy in day-care centres with a brief telephone intervention for informal caregivers will make it easier for the caregivers to reconcile the home care they are providing with their work, thus delaying the transition to institutional care and decreasing the costs for the healthcare system.

Research hypotheses:

i. The MAKS therapy leads to a statistically significantly greater improvement in abilities to perform ADL and cognitive capacities over time in people with MCI and dementia as compared with “care as usual” in the control group.

ii. The combination of MAKS therapy and brief telephone interventions for informal caregivers leads to statistically significantly more favourable subjective perceptions of burden and well-being in informal caregivers than in the control group.

iii. The interventions lead people with cognitive impairment to remain in home care for longer after the 2-year period of data collection and thus result in an economic health advantage over the control group.

### Study design and setting

A cluster-randomised, controlled, multicentre, prospective longitudinal study with a waitlist control group design was conducted to test the above research hypotheses.

**Fig. 1:**
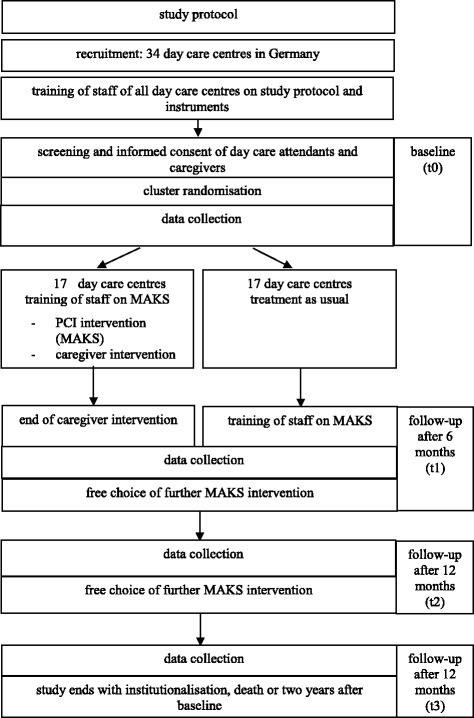
Study Design

All day-care centres participating in the study (cluster) were randomly assigned to the intervention or control group at baseline (random selection). The cluster was stratified by region (the Northeast, the Southeast, and the West). In order to guarantee that the sequence would be concealed until the intervention and control groups were assigned, all day-care centre staff were trained on the study protocol, instruments, and screening before the randomisation. The informed consent of the day-care users and their caregivers were also collected before the randomisation (see Fig. 1). Day-care centres were informed of their group allocation in written form by the study headquarters. During the 6-month intervention phase, the day-care centres in the intervention group carry out the MAKS treatment after their staff receive training. The caregivers receive a brief telephone intervention by counsellors with training in psychotherapy. The day-care centres in the control group do not carry out any additional project-specific treatment but continue providing “care as usual”. This is the accepted standard of care in day-care centres. Because we are investigating a non-pharmacological intervention, neither the staff carrying out the MAKS therapy nor the people with cognitive impairment are blinded. Six months after baseline, the day-care centres’ staff in the control group will also undergo training in MAKS therapy. At the end of the 6-month intervention phase, the day-care centres in both the intervention and control groups will freely decide whether or not to continue the MAKS treatment or to introduce the treatment, respectively, including the level of intensity. The observation period is 2 years with a total of four measurement points (baseline and after 6, 12, and 24 months). Data are collected by means of psychometric tests, the day-care centres’ documentation, and computer-assisted telephone interviews (CATIs) with the caregivers. All procedures were approved by the Friedrich Alexander University Erlangen-Nuremberg Ethics Committee. The external GKV Spitzenverband was closely informed of the progress of the study and achieved milestones as determined in the trial application. The study design is presented in Fig. 1. In the case of important protocol modifications, we will inform the Ethics Committee, the funders, the day-care centres, and the platform for the trial registry. Trial registration data are presented in Table 1.

**Table 1:** Trial registration data

Data category	Information
1. Primary registry and trial identification number	ISRCTN16412551
2. Date of registration in primary registry	30 July 2014
3. Secondary identifying numbers	GKV-SV201
4. Source(s) of monetary or material support	Long-Term Care Insurance Funds (GKV-Spitzenverband), German National Association of the Statutory Health Insurance and
5. Primary sponsor	Long-Term Care Insurance Funds (GKV-Spitzenverband), German National Association of the Statutory Health Insurance and
6. Secondary sponsor(s)	-
7. Contact for public queries	See point 8
8. Contact for scientific queries	Prof. Dr. Elmar Graessel, elmar.graessel@uk-erlangen.de PD Dr. Katharina Luttenberger, katharina.luttenberger@uk-erlangen.de
9. Public title	The German Day-Care Study
10. Scientific title	Multimodal non-drug therapy for persons with cognitive decline in day-care institutions with short-term interventions for informal caregivers by telephone to strengthen the compatibility of care and work
11. Countries of recruitment	Germany
12. Health condition(s) or problem(s) studied	Mild cognitive impairment, mild or moderate dementia (degenerative type, not solely vascular)
13. Intervention(s)	Intervention group: Activation therapy for day-care users with cognitive impairment
	Control group: usual care offered in each day care (treatment as usual)
	Informal caregiver: telephone intervention
14. Key inclusion and exclusion criteria	Ages eligible for study: adults; Sexes eligible for study: both
	Inclusion criteria:
	1. Users of day-care centres with mild cognitive impairment or early dementia who have an informal caregiver
	2. Informed consent
	Exclusion criteria:
	1. Completely blind or deaf
	2. No informal caregiver at all
	3. Severe dementia
	4. Cognitive decline due to diseases other than dementia (e.g. schizophrenia or Korsakov)
15. Study type	Cluster-randomised controlled intervention study
16. Date of first enrolment	October 2014
17. Target sample size	350
18. Recruitment status	Complete
19. Primary outcome(s)	Users of day-care centres: cognition (MMSE), activities of daily living (ETAM)
	Caregivers: subjective burden (HPS-K), well-being (WHO-5)
20. Key secondary outcomes	Users of day-care centres: e.g. NPI-Q, EQ-5D
	Caregivers: EQ-5D, BIZA-D

### Sample size estimation

A power analysis was computed on the basis of the authors’ previous experience with the MAKS study in nursing homes [15, 16, 22]. In contrast to the procedure in the MAKS study in nursing homes, the intervention in the DeTaMAKS project had a lower treatment intensity and a shorter duration, and therefore the effect was expected to be markedly lower. We conservatively set an expected effect size of f^2^ = 0.06 for the primary outcome and alpha error of 0.05 and a statistical power of 0.80 and obtained a total sample size of approximately 270 people attending a day-care centre for at least 6 months. Based on pilot study we assumed fluctuation in day-care centre users of 22% over 6 months. Therefore, we concluded that a total of 346 people should initially be included in the study.

The results of the MAKS study in nursing homes also showed that 69% of eligible candidates were willing to take part in the study. The pilot study showed that 56% of the day-care centre users who were screened fulfilled the required criteria for inclusion. This means that a total pool of 1086 day-care centre users was needed to achieve the required sample size. If we assumed approximately 33 users per week, we needed to recruit at least 32 day-care centres.

These day-care centres were large enough to have the space and staff required to carry out the project in accordance with the prescribed conditions.

### Recruitment strategies

Day-care centres from all regions of Germany were identified by means of their websites or entries in information systems and were contacted by telephone. If they met the required size (no fewer than 15 places) and expressed interest, they were sent basic written information about the project. A co-operation contract was signed with the day-care centres that decided to participate in the study. We stopped recruiting further day-care centres after the contract with the 34th day-care centre had been signed. Before the screening phase, all participating day-care centres were given one day of training by the study team on the study protocol (2 trainees per day-care centre) and one day on the recruitment instruments (1-2 trainees per day-care centre).

### Eligibility of participants

All users of the 34 day-care centres and their caregivers were included in the screening process. All dyads (consisting of a day-care centre user and caregiver) that fulfilled the criteria for inclusion were informed about the study and asked to take part in the project.

#### Day-care centre users

All day-care centre users underwent a two-step standardised screening process to determine their suitability for the project. In the first step of the screening process, all day-care centre users who had been documented by their day-care centre as fulfilling at least one of the following criteria were excluded: blindness, deafness, lacking a caregiver, lacking the ability to communicate, more than 1 stroke, severe depression, schizophrenia, an addictive disorder, concrete plans for institutionalisation, and attendance at the day-care centre of less than once a week. The day-care centres’ documentation contained all medical diagnoses and doctors’ prescriptions known to the informal caregivers. In a second step, the day-care centre users who remained after the first step of the screening process underwent psychometric testing of their cognitive performance.

The Mini-Mental State Examination (MMSE) and the Montreal Cognitive Assessment (MoCA) were administered in combination to screen the day-care centre users for MCI, mild dementia, and moderate dementia. The MMSE was administered first in order to differentiate between non-dementia and dementia on the basis of the cut-off score of 23 points [23]. However, as the MMSE is not sensitive enough to be able to detect MCI in the range of non-dementia cases [23–25], the MoCA was also administered when the MMSE values ranged from 24 to 30 points. Freitas [26] suggests using a cut-off score of 22 points to discriminate between normal cognition and MCI. The criteria for day-care centre users who were positively screened for MCI or mild to moderate dementia are shown in Table 2.

**Table 2:** Definition of positively screened day-care users

	Normal cognition	MCI	Mild or moderate dementia	Severe dementia
Step 1: MMSE	30-24	30-24	23-10	9-0
Step 2: MoCA^a^	30-23	22-0	-	-
Decision	Exclusion	Inclusion	Inclusion	Exclusion

^a^MoCA was applied only if the MMSE results were in the range of 24 to 30 points

The day-care centre users who consented to take part in the study after being given detailed personal and written standardised information were included in the final sample if their informal caregivers also gave consent. When the day-care centre user had a legal guardian, the guardian’s consent was also obtained. The heads of staff at the day-care centres collected the informed consent.

#### Caregivers

**Fig. 2:**
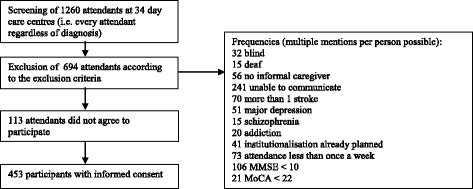
Flow Chart of the recruitment process

A main precondition for study participation was the existence of an informal caregiver who provided home care but was not an employee. When there were several informal caregivers, as a rule, the caregiver who currently was not yet retired was asked to take part in the study. The caregiver was not required to be either a relative or to live with the day-care centre user. Like the person with cognitive impairment, the caregiver was also thoroughly informed about the study. Figure 2 presents the development of the numbers of cases assessed over the entire screening process, at the end of which a sample of 453 dyads was included.

### Interventions

#### MAKS therapy: A multicomponent intervention for people with cognitive impairment

MAKS [14, 15, 22] consists of four components that are combined in the same order every day in a manualised intervention module lasting approximately 2 h. The participants have to use social skills in order to interact in a group and complete some of the tasks together. Thus, cooperation is promoted in all components.

#### The contents of the MAKS therapy

Table 3 provides an example of how the MAKS therapy is structured for 1 week. The daily treatment module begins with an introductory social warm-up session, lasting approximately 10 min. This can include, for example, a check-in session and a discussion about various subjects, meditation and perception, and sensory exercises. In the sensorimotor activation session that follows, lasting roughly 30 min, the domains of general mobility, gross and fine motor skills, balance, and sensory perception are practised, for example, through limbering up exercises, dance and sitting dance, movement games, fine motor exercises, and various kinds of games and sports (e.g. skittles, darts, etc.).

**Table 3:** Example of weekly plan for MAKS Therapy

One-week plan for MAKS	Social warm-up session 10 min	Sensorimotor activation training 30 min	Break 10 min	Cognitive training 30 Min	Training in activities of daily living 40 min
Monday	Thought-provoking impulse: what I am grateful for	Balance training		Projector exercise: finding pairs	Preparing a dessert with fruit, yoghurt, and pistachios
Tuesday	Breathing meditation	Table football		Projector exercise: putting picture stories in the right order	Making a key rack Part 1
Wednesday	Comparing the past and today: What does the season of spring mean to me? What did it mean in other life stages?	Towel exercises		Paper-and-pencil exercise: linking sentences together	Making a key rack Part 2
Thursday	Perception exercise: smelling; fruit	Playing darts with grain pillows		Projector exercise: finding shadows	Baking a cake
Friday	Provoking interaction: How did I feel when I arrived and how did I get here today?	Combining gross motor skills and fine motor skills: setting the table for tea		Projector exercise: “What goes together?” Café	Tea-time group with music and dancing

After a break, cognitive activation continues for an average of 30 min. Cognitive processes (especially memorising, recognising, forming associations, and language comprehension) and cultural techniques (reading, writing, arithmetic) are promoted through ten digitalised groups of cognitive tasks specially developed for the DeTaMAKS project with three different levels of difficulty (see Table 4), complemented by paper-and-pencil exercises.

**Table 4:** New digital cognitive exercises

Group of tasks	Explanation
Naming and linking	Naming objects, thinking of other objects in the same category, selecting an object whose name begins with a certain letter
Completing pictures	Saying what detail of a picture or object is missing that is necessary for it to work or should normally be there
Putting picture stories in order	Putting pictures with individual scenes on them in order so that a meaningful story is created
Memorising	Memorising a list of pictures, symbols, or words and recognising or reproducing them again
Finding pairs	Identifying the pictures in a set that fit together logically and creating meaningful pairs
Series exercise	Continuing a series of symbols in a logically correct way
Finding shadows	Identifying which shaded shape is the correct outline of which picture
Finding symbols	Recognising target symbols in the group of symbols presented
What goes together?	Choosing common objects that match certain places or scenes
Knowledge quiz	Answering general knowledge questions of varying difficulty levels by selecting the correct response

It is particularly important to include forms of activation with higher levels of difficulty to allow the participants with MCI to improve their skills [27, 28].

In addition to techniques that are immediately applicable in everyday life, the final 40-min session in which ADL are activated was designed to promote a wide range of skills, including gross and fine motor skills, mobility and social skills, but also cognition, especially procedural memory. Examples of the contents of these activity sessions are household activities (cooking, baking), shopwork (e.g. making a key rack), gardening (e.g. growing radishes, planting spring flowers), social activities (e.g. celebrating current festivals, for example, New Years or Carnival), and crafts (e.g. making greetings cards or table decorations) [14].

#### Implementation of the MAKS therapy in the DeTaMAKS project

The day-care centres in the intervention group committed themselves to carrying out the therapy in accordance with the manual every morning from Monday to Friday for 6 months. On each day of the therapy, all study participants attending the day-care centre on that day take part in the MAKS therapy. This results in average “doses of therapy” of one to five therapy days per week. The MAKS therapy is carried out by no less than two trained therapists. At least one of the two therapists is a qualified healthcare professional (geriatric nurse, nurse, or occupational therapist). The one-day training event for the staff of the day-care centres and the material used in the therapy were given to the day-care centres in the intervention group at baseline and will be given to the centres in the control group 6 months later. Study headquarters’ staff members are available to answer questions by phone or by e-mail on all weekdays.

The project does not exert any influence on the usual services of any of the 34 day-care centres, the pharmacotherapy of the study participants, or the frequency of attendance. Day-care users were free to choose their day-care centre and were free to decide whether to participate in the MAKS therapy. However, all changes in the frequency of attendance of the day-care centre are documented for all study participants.

A precursor to the intervention was implemented in nursing homes in a randomised-controlled trial [15] to record serious adverse events (falls resulting in injury, other types of serious injury and deaths), which did not increase over a period of 12 months. This is why we forwent the recording of serious adverse events (except death) and why no stopping guidelines were necessary. For this reason, we expect that the benefits of the MAKS therapy will far outweigh any possible harm. Also, because the intervention was implemented within the care of the day-care centres, participants did not need additional accident insurance.

#### Brief telephone intervention for informal caregivers

In addition to the MAKS therapy provided by the day-care centres, all caregivers in the intervention group receive three brief outreach telephone interventions. The task of the counsellors is to support the caregivers through three manualised phone calls, each lasting roughly 1 h. The goal is to work with the caregivers to develop strategies for self-management, to reduce the stress involved in providing home care, and to deal with challenging behaviours in order to “empower” the caregivers by improving their skills. The intervention includes tried and tested procedures from stress psychology, adjusted to fit the informal caregivers’ situations. Specifically, elements of the following procedures are employed: stress inoculation training as described by Meichenbaum [29], time structuring for the day, selection and prioritisation of goals, and development of positive activities. The procedure follows the self-management approach [30], that is, the aim is to enable the caregivers to use the strategies they have learned as fast as possible and to apply them in a beneficial manner.

“Challenging behaviours”, which occur in dementia in particular, are frequently experienced as especially stressful by caregivers. The intervention therefore includes information on the reasons for behaviour that is outside of the norm and the teaching of strategies that can be applied to reduce the occurrence of challenging behaviours, especially including references to the established Need-Driven Behaviour model to understand challenging behaviour [31, 32]. The counsellor’s basic attitude is client-centred [33] and solution-oriented.

The phone calls are made at the beginning of the 6-month intervention phase, after about 2 months, and towards the end of the intervention phase. The intervention is carried out by two counsellors with training in psychotherapy who were given special training beforehand and regular supervision. The first phone call begins by determining the specific stress triggers and the behaviours that the caregiver identifies as challenging, followed by an explanation of some initial possible coping modes and information about the challenging behaviours the caregiver has experienced. The telephone counsellors help the informal caregivers develop concrete changes in their own behaviour. When necessary, the counsellors suggest that the caregiver might wish to take advantage of support services in their area. In the second phone call, the behaviour changes proposed in the first phone call and their effects are discussed. The third phone call serves as a booster session and is made towards the end of the 6-month intervention phase. Jointly developed “homework” tasks are intended to support learning and to help caregivers work on their issues during the periods between the phone calls.

### Measures

The measures employed at the different measurement occasions are shown in Table 5.

**Table 5:**
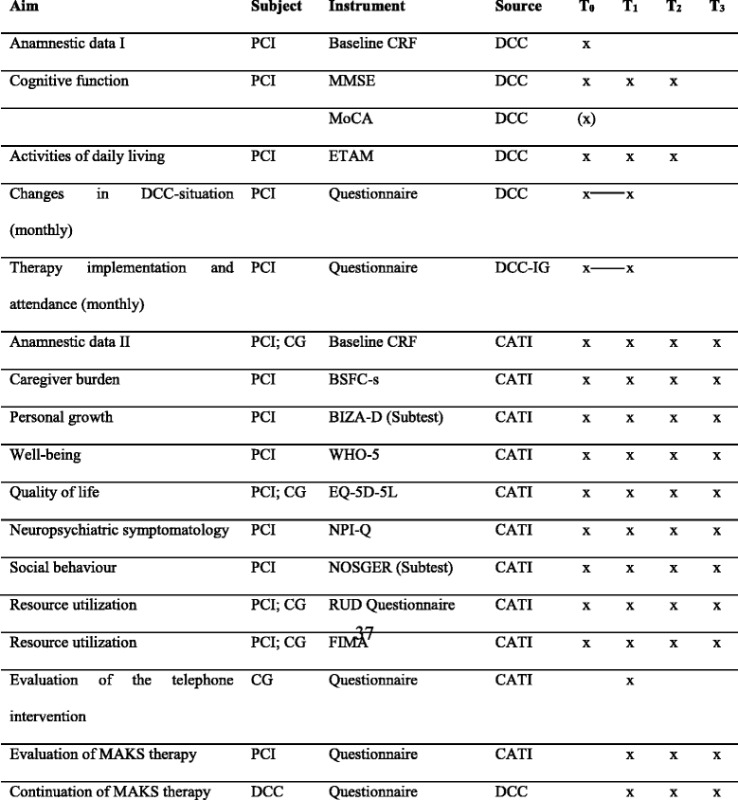
Timeline of measurements

*Abbreviations*: *BIZA-D* Berlin Inventory of caregivers’ burden with dementia patients, *BSFC-s* Burden Scale for Family Caregivers short, *CATI* computer assisted telephone interview, *CG* informal caregiver, *CRF* case report form, *DCC* day-care centre, *EQ-5D-5L* EuroQol five dimensions questionnaire, *ETAM* Erlangen test of activities of daily living in persons with mild dementia or mild cognitive impairment, *FIMA* Questionnaire for the use of medical and non-medical services in old age, *IG* intervention group, *MMSE* Mini-Mental State Examination, *MoCA* Montreal Cognitive Assessment, *NOSGER* Nurses’ Observation Scale for Geriatric Patients, *NPI-Q* Neuropsychiatric Inventory-Questionnaire, *PCI* person with cognitive impairment, *RUD* Resource Utilization in Dementia, *WHO-5* Well-Being Index

#### Primary outcome measures – People with cognitive impairment

Erlangen Test of Activities of Daily Living in Persons with Mild Dementia or Mild Cognitive Impairment (ETAM) [34]. The ETAM is a performance test for the assessment of the ability to perform ADL in people with mild dementia or mild cognitive impairment. It takes 19-35 *min* and consists of six items representing the five chapters of the domain “Activities and Participation” of the International Classification of Functioning, Disability, and Health (ICF). The score ranges from 0 to 30 points, with higher values showing better abilities to perform ADL. The ETAM meets the criteria for a well-constructed test [34].

*Mini-Mental State Examination (MMSE)* [35]. The MMSE is the most frequently employed screening test for dementia [36]. It measures five areas of cognitive functioning: orientation, registration, attention and calculation, recall, and language. The score ranges from 0 to 30 points, with higher scores representing better cognitive performance. Values above 23 points are interpreted as “not demented”. Scores between 0 and 23 are indicative of a dementia syndrome, more specifically, 18-23 points as “mild dementia”, 10-17 as “moderate dementia”, and 0-9 points as severe dementia [23].

#### Primary outcome measures – Caregivers

*Burden Scale for Family Caregivers short (BSFC-s)* [37]. The BSFC-s is used to assess the subjective burden of informal caregivers. The ten items of the short version are rated on a scale ranging from 0 (strongly disagree) to 3 (strongly agree). The total score ranges from 0 to 30 points, with higher values indicating a greater burden.

*WHO-5 Well-Being Index (WHO-5)* [38, 39]. The WHO-5 is employed to assess well-being during the last 14 days. Five positively poled statements are rated on a 6-point scale ranging from 0 (At no time) to 5 (All of the time). The score ranges from 0 to 25 points, with higher values indicating greater well-being.

#### Secondary outcome measures – People with cognitive impairment

*EuroQol five dimensions questionnaire (EQ-5D-5L)* [40]. The EQ-5D-5L assesses health-related quality of life. It consists of the five items mobility, self-care, usual activities, pain/discomfort, and anxiety/depression. Each item is rated on a five-point scale.

*Neuropsychiatric Inventory Questionnaire (NPI-Q)* [41]. The NPI-Q is an observer rating scale for the evaluation of neuropsychiatric symptoms by the informal caregiver covering the 12 symptom areas delusions, hallucinations, agitation/aggression, depression/dysphoria, anxiety, elation/euphoria, apathy/indifference, disinhibition, irritability/lability, motor disturbance, night time behaviours, appetite/eating. Each symptom is tested with a screening question (present yes/no). If the response to a screening question is “yes”, specific aspects (domains) are explored further. In this study, only the screening questions were asked.

*Nurses’ Observation Scale for Geriatric Patients (NOSGER)* [42]*.* The NOSGER is an observer rating scale for assessing general abnormalities in the elderly. It consists of six subscales (mood, disturbing behaviour, social behaviour, memory, ADL, and instrumental activities of daily living (IADL)). Each subscale contains 5 items. Each item is rated on a 5-point scale ranging from 1 (always) to 5 (never). The number of points that could be scored for each subscale ranges from 5 to 25, with higher values indicating greater impairment. In this study, we used the “social behaviour” subscale.

#### Secondary outcome measures – Caregivers

*EQ-5D-5L* [40]*.* The EQ-5D-5L is used to evaluate the caregivers’ health-related quality of life.

*Berlin Inventory of caregivers’ burden with dementia patients (Berliner Inventar zur Angehörigenbelastung – Demenz, BIZA-D)* [43]. The BIZA-D is employed to assess psychosocial impairment in caregivers arising from caregiving. The measure contains 88 items divided into 20 subscales. Only the “benefits” subscale is used in this study. This subscale contains 5 items that are rated on a five-point scale. The score ranges from 0 to 20 points, with higher values indicating more “benefits”.

#### Other measures

*Montreal Cognitive Assessment (MoCA)* [25]. The MoCA is a measure that is used to screen for MCI. It consists of more difficult items than the MMSE and is thus able to better detect MCI [24, 26, 44, 45]. The score ranges from 0 to 30 points, with higher scores indicating better cognitive performance. A score of 22 or less is indicative of cognitive impairment [26].

*Resource Utilization in Dementia (RUD) questionnaire* [46]*.* The RUD is the measure that is used the most around the world to collect data on the use of resources in dementia, enabling comparisons of costs of care across countries with different health care provisions. It evaluates formal and informal care, specifically the use of resources by both the people requiring care and their informal caregivers. The part of the questionnaire that refers to formal care was adjusted to fit the German healthcare system.

*Questionnaire for the use of medical and non-medical services in old age (Fragebogen zur Inanspruchnahme medizinischer und nicht-medizinischer Versorgungsleistungen im Alter, FIMA)* [47]*.* This questionnaire is used to collect data on the use of healthcare resources by elderly people. It assesses the quantities and time periods for the services that are used. In this study, we used the subscale “nursing and household care”, which asks about formal care in detail.

*Users’ personal and medical histories.* The following data from the users’ histories were recorded in standardised form by the staff of the day-care centres at the start of the study (baseline): sociodemographic data (age, sex), information about their care situations (date of first visit to the day-care centre and frequency of attendance, level of care, use of an outpatient homecare service), and medical data (diagnoses, medications). Caregivers were asked to provide information on family status, level of education, and the duration of the care situation for the user and for themselves. In order to be able to establish changes in the caregivers’ employment, living, and care situations, this information was recorded at each measurement occasion.

*Changes in the day-care centre situation (monthly)*. During the 6-month intervention phase, we kept track of the presence of the study participants. Specifically, we recorded periods of and reasons for extended absences (longer than 1 week), changes in the level of care, and whether the participants left the day-care centre (terminated the contract, moved to a nursing home, died) or the project (withdrew consent). These data were tracked daily and reported monthly.

*Evaluation of the MAKS therapy.* The MAKS therapists in the day-care centres in the intervention group documented the attendance of the study participants and any deviations from the manualised procedure on each day of therapy of the 6-month intervention period in order to monitor the intensity and quality of the MAKS therapy. After the end of the 6-month intervention period, the therapists at the day-care centres attended by the intervention group participants were also asked to submit their subjective assessments of the 3 most suitable and 3 least suitable tasks in each MAKS component. In addition, from the follow-up survey conducted after 6 months and onwards, the informal caregivers in both arms of the study were asked to rate potential changes in the care recipients’ alertness/activity, initiation of contact/talkativeness, physical mobility, mood, and thinking/memorising capacities on a 3-point scale (improved/remained the same/worse). They were also asked about the occurrence of three types of severe adverse events (falls resulting in injury, other injuries requiring medical treatment, and other serious adverse events).

In order to obtain information on the sustainability of the MAKS therapy 12 and 24 months after baseline, the day-care centres are asked how they are carrying out the MAKS treatment after the end of the 6-month intervention phase.

*Evaluation of the brief telephone intervention for informal caregivers.* After the end of the 6-month intervention phase, the caregivers in the intervention group were asked to evaluate the brief telephone intervention. The questionnaire contained 4 questions that pertained to satisfaction with the intervention (example: “I learned from the counselling how I can pay better attention to my own needs”) that were rated on a 3-point scale (“I totally agree”, “partly yes, partly no”, “I don’t agree at all”). They were also asked about qualitative aspects (“What did you like?”, “What did you dislike?”).

### Data collection

Data collection for the primary outcome variables pertaining to the people with cognitive impairment was conducted at the day-care centres by staff who were trained by the study headquarters staff. Data are collected at the beginning of the study, after the end of the 6-month intervention period, and after 12 months. To reduce the risk of investigator bias, the testers are not involved in the care of the day-care centre users.

The CATIs are administered to the caregivers by trained interviewers at the beginning of the study and after 6, 12, and 24 months in order to collect data on variables related to the caregivers’ situations (self-rating), the day-care users’ situations (observer rating), and the care situation. The procedure of CATI is described in detail by Holle et al. [48]. A short form of the questionnaire is sent to the caregivers to prepare them for the interview. All the other data mentioned in the previous section are collected by trained day-care centre staff in a standardised, written form.

The final data set consists of data collected at the day-care centres and via CATIs. The data from the day-care centres exist in written form sent to the study headquarters and are later imported electronically. The CATI data are collected and stored electronically. Some of the outcome measures (e.g. care level) are collected in both ways.

Because data collection for the primary outcome variables is conducted at the day-care centres, follow-up data on drop-outs due to death or transfer to a nursing home cannot be collected. If caregivers cannot be reached by telephone for the CATI after three tries, they are sent the questionnaires by mail with the request to mail the questionnaires back.

A pseudonym is created for the electronic data, and it cannot be used for user identification. Only the key file contains information about a person’s identity and pseudonym. All of the data (including written documents, key files, etc.) are kept securely in a locked file, and only the researchers have access to the data.

### Data quality management

All of the groups involved in the study (MAKS therapists, counsellors for the informal caregivers, testers, CATI interviewers, and monitors at the day-care centres) are thoroughly trained for their respective tasks by the study headquarters staff. When they have questions, the members of all groups could contact the study headquarters at any time by phone or in writing.

In order to ensure the validity of the data, the data sources (tests, CATIs, day-care centres) are subjected to a random internal audit. To obtain evidence of the inter-rater reliability of the ETAM test and the CATIs, 5% of the baseline data were collected with the participation of a second person who is there to observe. Four (24%) of the day-care centres in the intervention group will be visited by personnel from the study headquarters during the first 6 months to monitor how the MAKS therapy is conducted (treatment adherence), the quality of the documentation (deviations from the manual and extraordinary events), and the attendance of the study participants.

The quality of the data is guaranteed by strict data monitoring at the study headquarters over the entire period of data collection. In addition, as some of the data are collected in two ways (e.g. care level) at the day-care centre and via a CATI with the caregiver, independent source document verification will be performed. Plausibility checks and logical considerations about the relationships between associated variables will be performed. Regular backups will be carried out and saved on an external hard drive.

### Analysis

#### Data analysis

Only researchers at the study centre in collaboration with the Institute of Health Economics and Health Care Management will assess the results. Our cooperation partner will check the plausibility of the data independently. Thus, there is no need for a data monitoring committee.

The data analyses will be performed with the aid of the “IBM SPSS Statistics 21” software.

If at least 80% of the items on a test have been answered, we will impute missing values from the mean score of the existing items. However, if more than 20% of the items on a test are missing for any given person (e.g. because a participant refuses to complete the test), we will compute the score according to the expectation maximum (EM) algorithm, which uses the variables that explain the greatest proportion of variance in the missing variable.

All changes to the data due to checking for errors, data quality management, or the handling of missing values will be documented.

In order to be able to assess the quality of the randomisation, the baseline data from the intervention and control groups will be examined for statistically significant differences.

The two hypotheses on the efficacy of the interventions in people with cognitive impairment and their caregivers will be tested by computing multiple regression analyses. The transition to institutionalised care as an event will be represented by a Kaplan-Meier curve for the intervention and control groups. The hypothesis on the transition to institutionalised care will be evaluated by Cox regression analysis.

As the primary outcome variables cannot be tested at follow-up in participants who dropped out of the study, the primary data analysis strategy will be “as treated”. The level of statistical significance will be set at *p* = 0.05. Results with an alpha error of less than 10% will be classified as statistical trends.

Subgroups will also be analysed exploratively, sub-divided in accordance with the severity of cognitive impairment and the mean frequency of attendance at the day-care centre per week.

#### Economic evaluation/ analysis

The valuation of resource use will be based on the valuation rates by Bock et al. [49]. The costs of the intervention include the training time and supervision of day-care centre staff, and materials (manuals, handbooks, etc.)., To analyse cost difference between groups, a multivariable model with a gamma distribution will be used to account for the skewed distribution of the data [50], adjusted for baseline costs. A 95-% confidence interval for the adjusted cost difference will be estimated from 1000 bootstrap replications. A cost-utility analysis from a societal perspective will be performed in which the quality of life of both day-care users and informal caregivers will be assessed. To calculate the quality-adjusted life years for day-care users regarding the time remaining at home, the quality of life values will be set to 0 from the day of admission to a nursing home or death. Moreover, a budget impact analysis will be computed to address changes in the expenses of the health care system if the MAKS therapy shows significant effects.

## Discussion

In this article, we describe the design of a large cluster-randomised, controlled study on the effects of a multicomponent intervention for people with cognitive impairment carried out at day-care centres. The effects on the users’ informal caregivers who receive a brief telephone intervention are also investigated.

Two conditions must be fulfilled to allow people with cognitive impairment to remain at home, which is desirable from both an economic perspective and the perspectives of the people with cognitive impairment and their caregivers. People with cognitive impairment need to sustain their cognitive capacities and their abilities to perform activities of daily living at as high a level as possible [9]. Their informal caregivers need to learn how to deal with the situation in a helpful way so as to keep their physical and mental burden as low as possible [10, 18]. This study addresses both of these conditions, first by providing an evaluated multimodal intervention for day-care centre users and second by supporting their caregivers through counselling that is oriented specifically towards caring for people with cognitive impairment.

The day-care centres have the advantage that they are in a position to combine two helpful approaches. Time is freed up for the caregivers, and the people with cognitive impairment can be offered an effective, non-drug group treatment.

### Strengths and limitations of the study design

Strengths of the study design are the cluster randomisation, the long follow-up period of 2 years, the use of a manualised treatment that has already been evaluated in nursing homes [15, 16, 22], the naturalistic setting in care institutions which are already part of the German health care system, and the focus on the dyad consisting of both the person with cognitive impairment and his/her informal caregiver.

Moreover, due to the recruitment of day-care centres throughout Germany and the balanced ratio of urban to rural day-care centres run by a wide variety of organisations (local authorities, non-profit, and private), the external validity of the study is high. The naturalistic design – in which the study has no influence on the number of days the people with cognitive impairment attend the day-care centres (days of treatment in the intervention group) – also makes it possible to analyse subgroups in order to determine the necessary intensity of the treatment.

We decided on cluster randomisation rather than randomisation on the participant level for two reasons. If two groups that are separate in terms of rooms and staff – one treatment group and one control group – were to take place at the same day-care centre, only a very few very large day-care centres would have the necessary capacity. These day-care centres are not representative of the general healthcare situation and would thus markedly reduce the external validity. In addition, implementing two study groups simultaneously in the same institution would be associated with a risk of halo effects by which the treatment might influence the control group.

The possible disadvantage of a systematic difference between the arms of the study arising by chance from the cluster randomisation is offset both by the large number of 34 randomised day-care centres and by the stratification by region [51].

Overall, it can be assumed that the control group consists of day-care centres that tend to be quite active and not only accept the extra work involved in the study but also receive the study-specific therapy 6 months after baseline. Differences between the groups will therefore tend to be under-estimated rather than over-estimated. Since the study requires extra work by the day-care centres, offering the treatment to the control group is necessary to motivate them to participate in the study in the first place. As the control group was also trained in the MAKS treatment after a delay of 6 months but did not get the supervision that the intervention group did, important hints for implementation can be drawn from the comparison between the two “intervention” phases, that is, between the regular, controlled intervention phase in the intervention group during the first 6 months and the less structured, independent implementation in the control group, which begins after 6 months of waiting.

In order to be able to replicate and implement any treatment, a good manual is needed, particularly in the case of non-drug therapies [52]. For the MAKS therapy, in particular, this is ensured by the compilation of 125 structured days of therapy in the 6-month controlled intervention phase. For each day of the study, the contents of each of the four MAKS components are carried out as stated in the study manual by all day-care centres. A manual was also created for the intervention for the caregivers. This makes both treatments easy to implement because only one training is required, and thus the necessary personnel and space are usually present on site.

One basic problem with non-drug therapies is the fact that they cannot usually be blinded. We try to protect the collection of the data on the primary outcomes for the day-care centre users as much as possible from a data collection bias. The trained testers are not allowed to be involved in the care of the day-care centre users and have to come from outside the centres to carry out their tasks.

As far as we know, this is the first time that the effectiveness of an evaluated non-drug therapy has been tested in people with cognitive impairment (including some with diagnosed dementia) in combination with outreach counselling for their caregivers in a sample in a real healthcare environment.

## Abbreviations

ADL: Activities of daily living
BIZA-D: Berlin Inventory of caregivers’ burden with dementia patients
BSFC-s: Burden Scale for Family Caregivers short
CATI: Computer-assisted telephone interview
DeTaMAKS: a cluster-randomised, controlled intervention study examining MAKS therapy for people with cognitive impairment in day-care centres
EQ-5D-5L: EuroQol five dimensions questionnaire
ETAM: Erlangen test of activities of daily living in people with mild dementia or mild cognitive impairment
FIMA: Questionnaire for the use of medical and non-medical services in old age
IADL: Instrumental activities of daily living
ICF: International Classification of Functioning, Disability, and Health
MAKS: Multicomponent group therapy for people with cognitive impairment (motor stimulation, activities of daily living and cognitive stimulation in a social setting)
MMSE: Mini-mental state examination
MoCA: Montreal Cognitive Assessment
NOSGER: Nurses’ Observation Scale for Geriatric Patients
NPI-Q: Neuropsychiatric Inventory-Questionnaire
RUD: Resource Utilization in Dementia
WHO-5: Well-Being Index
